# Relationships of Pain Treatment With Dementia and Functional Outcome in Medicare Home Health Care

**DOI:** 10.1093/geroni/igab046.631

**Published:** 2021-12-17

**Authors:** Jinjiao Wang, Kenrick Cato, Yeates Conwell, Kathi Heffner, Fang Yu, Thomas Caprio, Yue Li

**Affiliations:** 1 University of Rochester, Rochester, New York, United States; 2 Columbia University, New York, New York, United States; 3 University of Rochester Medical Center, Rochester, New York, United States; 4 Arizona State University, Phoenix, Arizona, United States

## Abstract

Adequate pain management is important to post-acute care functional recovery, yet persons with Alzheimer’s disease and related dementias (ADRD) are often under-treated for pain. The objectives of this study were to examine in Medicare post-acute home health (HH) recipients with daily interfering pain 1) if analgesic use at home is related to functional outcome, and 2) if ADRD is related to the likelihood of analgesic use at home. We analyzed data from the Outcome and Assessment Information Set, Medicare claims, and electronic medical records of 6,039 Medicare beneficiaries ≥ 65 years who received care from a large HH agency in New York in 2019 and reported daily interfering pain. Analgesic use was identified in medication reconciliation of HH visits and categorized into any analgesics or opioid(s). ADRD was identified from ICD-10 codes and significant cognitive impairment. Functional outcome was measured as change in the composite score of Activity of Daily Living (ADL) limitations from HH admission to HH discharge. Use of any analgesics at home was associated with greater ADL improvement from HH admission to HH discharge (β= -0.20 [greater improvement by 0.2 ADLs], 95% Confidence Interval [CI]: -0.37, -0.04; p=0.017). Compared with patients without ADRD, those with ADRD were less likely to use any analgesics (Odds Ratio [OR] = 0.66, 95% CI: 0.49, 0.90, p=0.008) or opioids (OR=0.53, 95% CI: 0.47, 0.62, p<0.001) at home. Adequate pain management is essential to functional improvement in post-acute HH care. Patients with ADRD may be under-treated for pain in post-acute HH care.

